# Macrophage activation drives ovarian failure and masculinization in zebrafish

**DOI:** 10.1126/sciadv.adg7488

**Published:** 2023-11-22

**Authors:** Paloma Bravo, Yulong Liu, Bruce W. Draper, Florence L. Marlow

**Affiliations:** ^1^ Department of Cell, Developmental and Regenerative Biology, Icahn School of Medicine at Mount Sinai, New York, NY, USA.; ^2^ Department of Molecular and Cellular Biology, University of California, Davis, CA, USA.

## Abstract

BMP15 is a conserved regulator of ovarian development and maintenance in vertebrates. In humans, premature ovarian insufficiency is caused by autoimmunity and genetic factors, including mutation of BMP15. The cellular mechanisms underlying ovarian failure caused by BMP15 mutation and immune contributions are not understood. Using zebrafish, we established a causal link between macrophage activation and ovarian failure, which, in zebrafish, causes sex reversal. We define a germline-soma signaling axis that activates macrophages and drives ovarian failure and female-to-male sex reversal. Germline loss of zebrafish Bmp15 impairs oogenesis and initiates this cascade. Single-cell RNA sequencing and genetic analyses implicate ovarian somatic cells that express conserved macrophage-activating ligands as mediators of ovarian failure and sex reversal. Genetic ablation of macrophages or elimination of Csf1Rb ligands, Il34 or Csf1a, delays or blocks premature oocyte loss and sex reversal. The axis identified here provides insight into the cells and pathways governing oocyte and ovary maintenance and potential therapeutic targets to preserve female fertility.

## Teaser

Bmp15 deficiency induces sex reversal in a macrophage and Csf1a dependent manner.

## INTRODUCTION

In many animals, including mammals, embryos initially develop with indeterminant gonads that differentiate into ovaries or testis when sex is determined. In zebrafish, the first gonads that form are ovary-like, including those that will ultimately develop as testis. In most males, the juvenile ovary undergoes a process of gonadal transformation before forming a testis. In females, the juvenile ovary further differentiates into a mature ovary. As in mammals, the zebrafish ovary is composed of germline cells and the supporting follicle cells of the somatic gonad—granulosa and theca cells—that support differentiation, growth, and maintenance of the germline. Although the zebrafish ovary is stably maintained in normal conditions, it can be remodeled to form a functional testis when oogenesis is compromised (*1*–*8*). Despite being a major vertebrate developmental model, neither the initial triggers of ovary or testis differentiation nor the ensuing mechanisms that regulate sexual differentiation of the juvenile zebrafish gonad into mature ovary or its transformation into testis are known (*9*). Transformation of the juvenile ovary to testis during development and the transition from mature ovary to testis associated with ovarian failure in adult zebrafish both involve elimination of oocytes and ovary tissues and replacement or remodeling of the germline and somatic gonad into testis. Despite these common features, neither the mechanistic basis of ovary-to-testis transition during development nor transitions resulting from ovarian dysfunction in adults are understood.

In humans, *bone morphogenetic protein 15* (*BMP15*) is a key regulator of ovarian development (*10*, *11*). BMP15, a conserved ligand produced by early oocytes of vertebrates, is an essential regulator of follicular growth that, in mammals, promotes granulosa cell proliferation (*12*–*14*). BMP15 secreted by oocytes binds bone morphogenetic protein receptor type 2 (BMPR2) and signals through SMAD1 to activate targets in the somatic gonad. In mouse, ovarian follicles with high BMP15 expression progress, while those with low levels undergo atresia (*15*). Accordingly, BMP15 has been implicated in the pathophysiology of premature ovarian insufficiency or failure (POI and POF, respectively) (*11*, *16*), which can involve a combination of genetic, endocrine, immune, and environmental factors. Variants in the *BMP15* gene are a predominant genetic cause of POI, a reproductive disorder caused by genetic and immunity-related disorders that affects oocyte quality and leads to hyperandrogenism and inflammation. POI and POF not only lead to sterility but also, more broadly, can adversely affect health, including bone, cardiovascular, and neurological pathologies (*17*). In zebrafish, Bmp15 is also essential for maintenance of the female germline. *bmp15* mutants initially develop as females but undergo ovarian failure and sex reversal, presumably due to granulosa cell and estrogen deficiencies (*7*). We showed that loss of oocytes but not follicle progression in *bmp15* mutants is attenuated by eliminating the conserved masculinizing transcription factor, doublesex- and mab-3–related transcription factor 1 (Dmrt1) (*18*). However, the underlying mechanisms and cellular mediators driving ovarian failure and sex reversal are not understood.

## RESULTS

### Bmp15 functions in follicle progression, ovary maintenance, and fertility

**Fig. 1.**
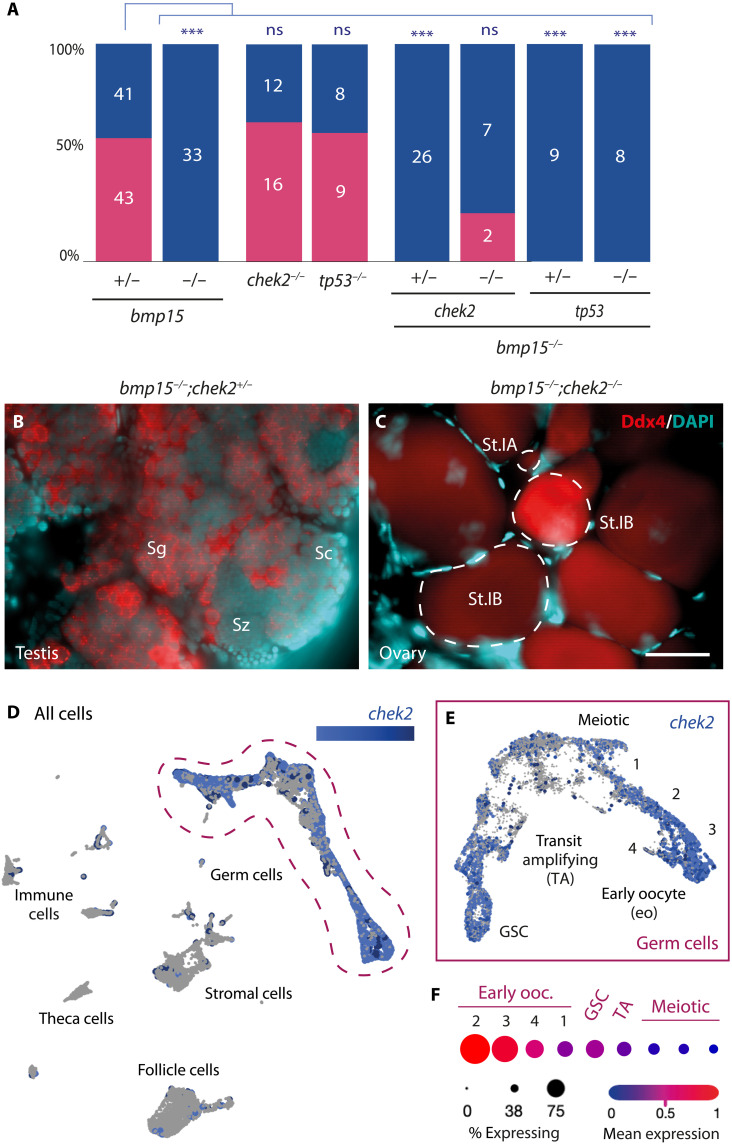
Loss of Chek2 suppresses oocyte death and sex reversal in the absence of Bmp15. (**A**) Adult sex ratios of indicated genotypes. Female, pink; male, blue. Numbers indicate individuals examined. Statistical analysis: chi-square test with Bonferroni correction; *P* value comparisons to *bmp15*^+/−^, ***P ≤ 0.0001. ns, not significant. (**B** and **C**) Immunostained adult *bmp15;chek2* gonads of indicated genotypes. Ddx4 (red) labels germ cells, and 4′,6-diamidino-2-phenylindole (DAPI; cyan) labels DNA. Scale bar, 50 μm. Sg, spermatogonia; Sc, spermatocyte; Sz, spermatozoa; St.IA, stage IA oocyte (prophase I meiotic cells in nests), St.IB, stage IB oocyte (late prophase within definitive follicles). (**D** and **E**) UMAP plot of *chek2* expression in the 40-dpf ovary in (D) all cells, and (E) reclustered germ cells. (**F**) Analysis of *chek2* expression profile in specific germ cells subclusters represented by dot plot graph. GSC, germline stem cells; TA, transit amplifying.

Although Bmp15 was known to be essential for ovary maintenance, this could be due to failure to promote Bmp15-dependent somatic cell fates, deficits of paracrine or autocrine factors, or a combination of these functions. To determine whether Bmp15 promotes follicle survival, we generated double mutants (DMs) lacking both Bmp15 and tumor suppressor factors (TSFs) tumor protein 53 (Tp53) or checkpoint kinase 2 (Chek2), which can suppress cell death and oocyte loss in some infertile zebrafish and mouse mutants (*19*–*21*). We found that, as in wild type, *bmp15* heterozygotes and *chek2* or *tp53* single mutants showed no sex bias by 85 days postfertilization (dpf), but all *bmp15* homozygous mutants were fertile males (Fig. 1A and fig. S1, A and B). Similarly, *bmp15* mutants that were also heterozygous for *tp53* or *chek2* and *bmp15;tp53DMs* (*n* = 8) were all male (Fig. 1, A and B, and fig. S1C). In contrast, two of the nine *bmp15;chek2DM*s retained oocytes through stage II (previtellogenic) and showed female secondary sex traits, including body coloration, fin morphology, and urogenital papilla structures at 95 dpf (Fig. 1, A and C, and fig. S1D). Notably, using previously reported single-cell RNA sequencing (scRNA-seq) data (*22*), we found that *chek2* is expressed predominantly in oocytes (Fig. 1, D to F). Therefore, we conclude that sex reversal of *bmp15* mutants, including primary and secondary sex traits, can be suppressed by preventing Chek2-mediated cell death of oocytes. However, suppression was only partially penetrant, and mutant follicles did not progress developmentally even when oocyte loss and ovary-to-testis sex reversal were blocked. This result indicates that Bmp15 signaling functions extend beyond survival and include regulating follicle progression. Therefore, Bmp15 has a conserved function in signaling from the oocyte to promote growth, survival, and progression of ovarian follicles, which prevents ovarian failure in zebrafish.

### Macrophages and ovary-to-testis transition

**Fig. 2.**
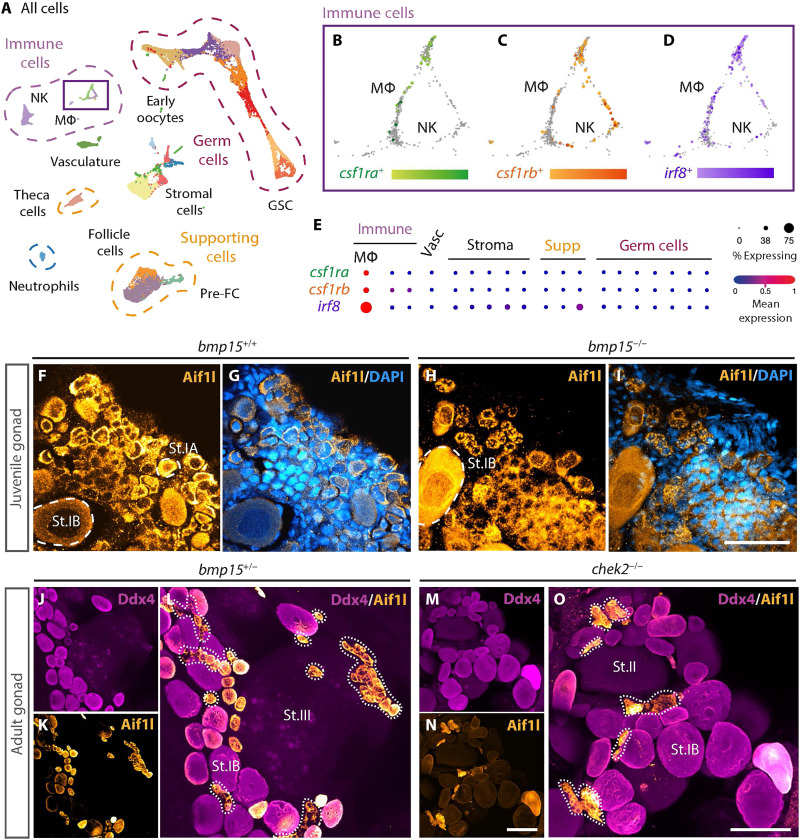
Macrophages are resident ovary cells in juvenile and adult zebrafish ovaries. (**A**) UMAP plot of cell types present in 40-dpf ovary. (**B** to **D**) Magnified view of populations boxed in (A) showing expression of indicated genes. MΦ, macrophages; NK, natural killer–like cells; FC, follicle cells; GSC, germline stem cells; Vasc, vasculature. (**E**) Analysis of indicated macrophage gene expression profiles in ovarian clusters represented by dot plot graph. (**F** to **O**) Immunostained (F) to (I) juvenile and (J) to (O) adult gonads of indicated genotypes. Aif1l (yellow) labels macrophages, Ddx4 (magenta) labels germ cells, and DAPI (blue) labels DNA. Dotted lines mark macrophages. Scale bars, (F) to (I) 50 μm and (J) to (L) 100 μm. St.II, stage II oocyte (apparent cortical alveoli and a vitelline envelope); St.III, stage III oocyte (increased size and apparent yolk).

Because preventing oocyte loss only partially suppressed sex reversal, we investigated potential involvement of somatic gonad populations in sex reversal/oocyte maintenance. Because immunity-related disorders are implicated in ovarian failure (*23*, *24*), we investigated the role of macrophages, which are key innate immune cells that eliminate foreign or dying cells from tissues. They regulate cell removal, produce cytokines and factors that recruit and regulate other immune cells, express growth factors to control proliferation and vascularization, and deposit extracellular matrix during wound healing and repair and thus contribute to tissue homeostasis (*25*–*27*). In mammalian ovary, macrophages are abundant and heterogeneous, showing distinct and ovarian cycle–dependent distributions and activation states (*28*, *29*). Furthermore, they are hypothesized to degrade the follicle by promoting granulosa cell apoptosis (*30*–*33*). scRNA-seq of 40-dpf ovary confirmed the presence of macrophages based on RNA expression of the zebrafish *colony-stimulating factor 1 receptors* (*csf1ra/b*) (Fig. 2, A to C and E, and fig. S2, A, B, and D) and *interferon transcription factor 8* (*irf8*), a conserved regulator of macrophage differentiation (Fig. 2, A, D, and E, and fig. S2, C and D) (*22*). Analyses of gene expression correlations among these genes and markers of other immune cell types, e.g., natural killer–like cells, confirm that *csf1ra/b* and *irf8* are expressed by macrophages (fig. S2E).

To determine whether loss of Bmp15 was associated with an increase in macrophages, we examined the inflammatory macrophage marker *aif1l* (*allograft inflammatory factor 1–like*, formerly called *iba1*) in 40-dpf wild-type and *bmp15-*mutant ovaries (*34*, *35*). Aif1l-positive cells included somatic cells and early oocytes in 40-dpf wild type (Fig. 2, F and G). Aif1l had a similar distribution in 40-dpf *bmp15-*mutant ovaries (Fig. 2, H and I). Juvenile ovaries at 40 dpf were chosen because this stage aligns with the scRNA-seq dataset and is a stage before ovary-to-testis reversal occurs in *bmp15* mutants. Furthermore, 40-dpf ovaries with stage II oocytes were selected as juvenile ovaries lacking stage II oocytes have the potential to undergo a late transformation to a testis and thus may not be future females. To determine whether suppression of oocyte loss in *chek2* mutants was due to loss of macrophages, we examined Aif1l, the macrophage marker 4c4 (*36*) and Ddx4, to mark the germline and confirm that the somatic cells were macrophages, in adult wild-type and *chek2-*mutant ovaries (Fig. 2, J to O, and fig. S2, H to L). That *chek2* is expressed in oocytes and that macrophages were present in wild-type and *chek2*-mutant ovaries suggest that Chek2 loss likely suppresses sex reversal of *bmp15;chek2DMs* by acting in oocytes to prevent their death rather than regulating macrophages. However, on the basis of this analysis alone, a potential role for activating or mobilizing macrophages in the absence of Bmp15 cannot be excluded.

**Fig. 3.**
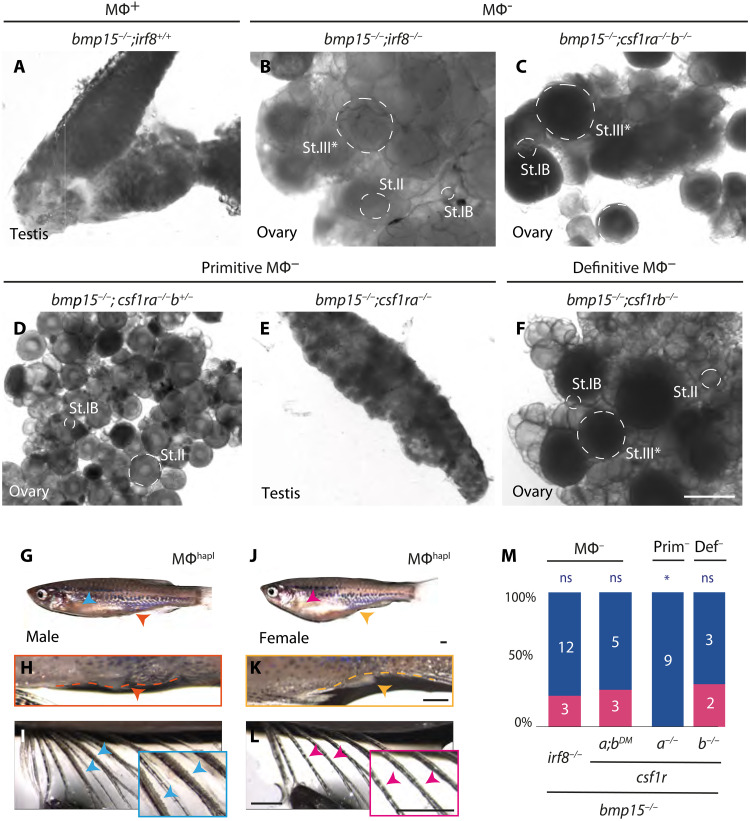
Definitive macrophages are required for ovarian failure and sex reversal of *bmp15* mutants. (**A** to **F**) Live tissue pictures of adult gonads of *bmp15* mutants when macrophages are (A) present, (B) and (C) completely ablated, (D) haploinsufficiency of, or lack of (E) primitive, or (F) definitive populations. Scale bar, 500 μm. St.III*, arrested stage III oocyte (increased size and apparent yolk). (**G** to **L**) Secondary sex traits of adult: (G) male body shape, (H) urogenital papilla (orange dashed line and arrowhead), and (I) lateral fin tubercles (blue arrowheads and box); (J) rounded female body shape, (K) distinct urogenital papilla (yellow dashed line and arrowhead), and (L) absence of tubercles (pink arrowheads and box). Scale bars, 1 mm. (**M**) Adult sex ratios of indicated genotypes. Female, pink; male, blue. Numbers indicate individuals examined. Statistical analysis: chi-square test with Bonferroni correction; *P* value comparisons to *bmp15*^+/−^, *P ≤ 0.0125.

To investigate whether macrophages contribute to follicle atresia and sex reversal, we genetically ablated macrophages in *bmp15* mutants using two genetic manipulations. In zebrafish, macrophages arise in two waves of specification and colonization. Loss of colony-stimulating factor 1 receptor a (Csf1ra) ablates primitive macrophages, which arise from the anterior lateral plate mesoderm in the early embryo ~16 hours postfertilization. By contrast, *csf1 receptor b* (*csf1rb*) mutation affects the definitive population that originates from the ventral dorsal aorta at 14 dpf (*37*, *38*). Despite distinct contributions to the primitive and definitive waves, both *csf1ra* and *csf1rb* single mutants have macrophage deficits as adults and DM fish lacking both receptors (*csf1r^DM^*) show a lifelong absence of macrophages without affecting sex ratios (fig. S2, F and G) (*39*–*41*). Similarly, mutation of *irf8* ablates macrophages without disrupting normal sexual differentiation (fig. S2F) (*42*–*44*). To study macrophage contributions to follicle atresia and sex reversal, we examined *bmp15* mutants lacking all macrophages (Mϕ^−^): *csf1r^DM^* or *irf8* mutants (Fig. 3 and fig. S3). Analysis of adult gonads revealed that *bmp15* mutants with macrophages were all fertile males, while mutant females lacking macrophages retained ovaries but were sterile. This is because *bmp15-*mutant oocytes arrest development before vitellogenic stages of oogenesis, and eliminating macrophages does not restore deficits in Bmp15-dependent somatic gonadal cell populations or related signaling between germline and soma. Instead, macrophages likely eliminate ovarian tissue and promote or facilitate gonad remodeling and development of testis tissue during ovarian failure. Consequently, suppression of sex reversal is incomplete, and mutant females remain infertile (Fig. 3, A to C and M, and fig. S3A).

Primitive macrophages are present in the early embryo before sex determination, are dependent on Csf1Ra, and are normally still present when ovarian failure occurs in *bmp15* mutants, whereas definitive macrophage populations, which are present during sex determination and through adulthood are dependent on Csf1Rb (*39*–*41*). Taking advantage of the duplicated *csf1Rs*, which are differentially required for primitive and definitive macrophage populations (fig. S2G), allowed us to assess the contribution of each macrophage population and to effectively deplete or remove macrophages at the onset of ovarian failure in *bmp15* mutants. Thus, we examined mutant fish deficient for primitive and/or definitive macrophages (Mϕ^haploinsufficiency^), e.g., homozygous mutant for one *csf1* receptor and heterozygous for the other or fish with mutant *irf8* alleles (Fig. 3, D to M, and fig. S3, B to F). We found that *csf1ra* mutants heterozygous for *csf1rb*, which eliminates primitive and most definitive macrophages, prevented ovarian failure and sex reversal of *bmp15* mutants (Fig. 3, D and G to M). Double heterozygosity for both *csf1* receptors (*csf1r^DH^*) or for *irf8* also prevented oocyte loss and sex reversal (fig. S3, D to F). These observations indicate that a threshold number or specific macrophage population mediates sex reversal.

**Fig. 4.**
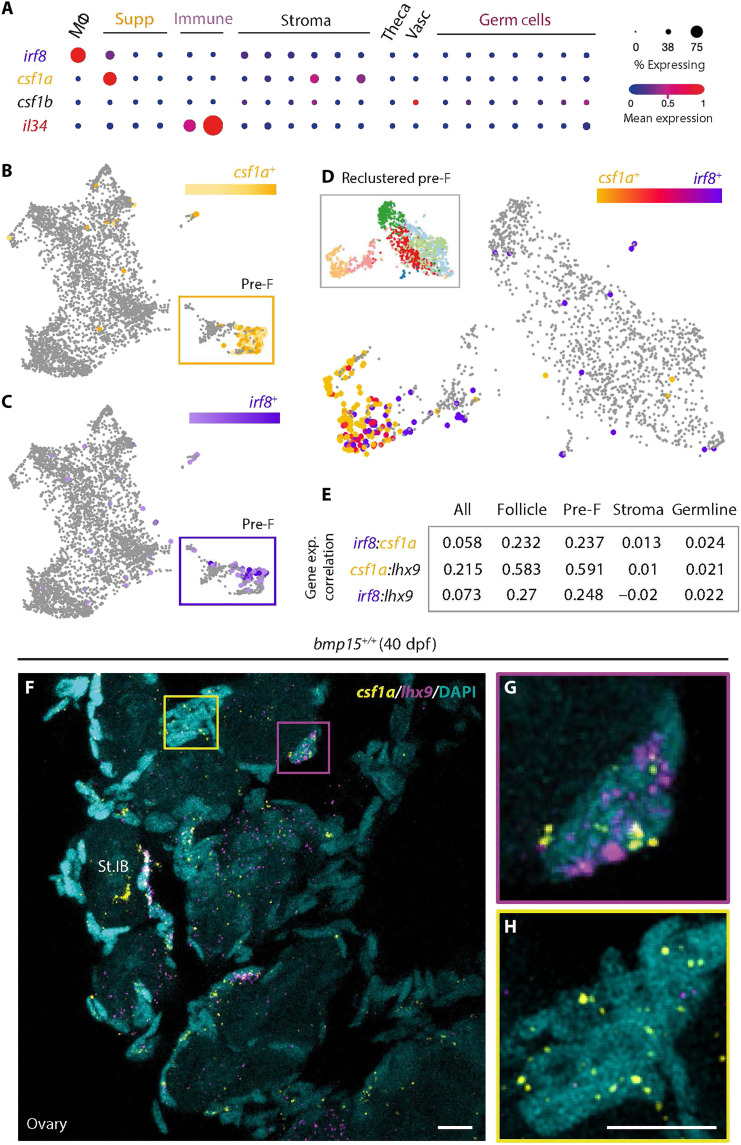
*irf8* and *csf1a* are coexpressed in a subpopulation of pre-follicle cells in the early ovary. (**A**) Analysis of expression profiles of indicated genes in specified clusters of ovarian cells represented by dot plot graph. (**B** to **D**) UMAP plot of indicated gene’s expression in the 40-dpf ovary in (B) and (C) follicle cells, and (D) reclustered subpopulation of pre-follicle cells. Subpopulation of pre-F cells boxed in (B) and (C). UMAP of reclustered pre-F cells; legend boxed in (D). (**E**) Gene expression correlation values of indicated genes in specified clusters of ovarian cells represented by Spearman’s Rho correlation analysis. (**F** to **H**) Double HCR RNA FISH confocal images of 40-dpf wild-type ovary. *lhx9*, follicle cells (magenta); *csf1a*, Csf1rs’ ligand (yellow), and DAPI, DNA (cyan). Regions boxed in (F) show magnified views of (G) *lhx9/csf1a-*coexpressing MAFCs and (H) *csf1a*-expressing somatic cells. Scale bars, 10 μm. F, follicle cells; Supp, supporting cells.

To determine whether ovarian failure and sex reversal requires a threshold number or a unique subtype of macrophages, we analyzed compound mutants for various combinations of *csf1ra/b* and found that *bmp15* mutants lacking only *csf1ra* were all male as adults (Fig. 3, E and M). Thus, ovarian failure and sex reversal occur in the absence of primitive macrophages. In contrast, loss or haploinsufficiency of definitive macrophages (Fig. 3, D and F to M, and fig. S3B) suppressed ovarian failure and sex reversal. In addition to macrophages, *irf8* was also expressed in stromal cells and some pre-follicle cells (Fig. 4, A and C to E). Nonetheless, we conclude that macrophages are direct cellular mediators of sex reversal in the absence of Bmp15, based on the genetic evidence that loss of *csf1ra/b*, which are highly expressed in macrophages, and loss of *irf8* both block sex reversal. Furthermore, sex reversal requires an activity unique to a specific state or subpopulation of definitive macrophages.

### Csf1 and Il34 ligand involvement in ovary-to-testis transition

**Fig. 5.**
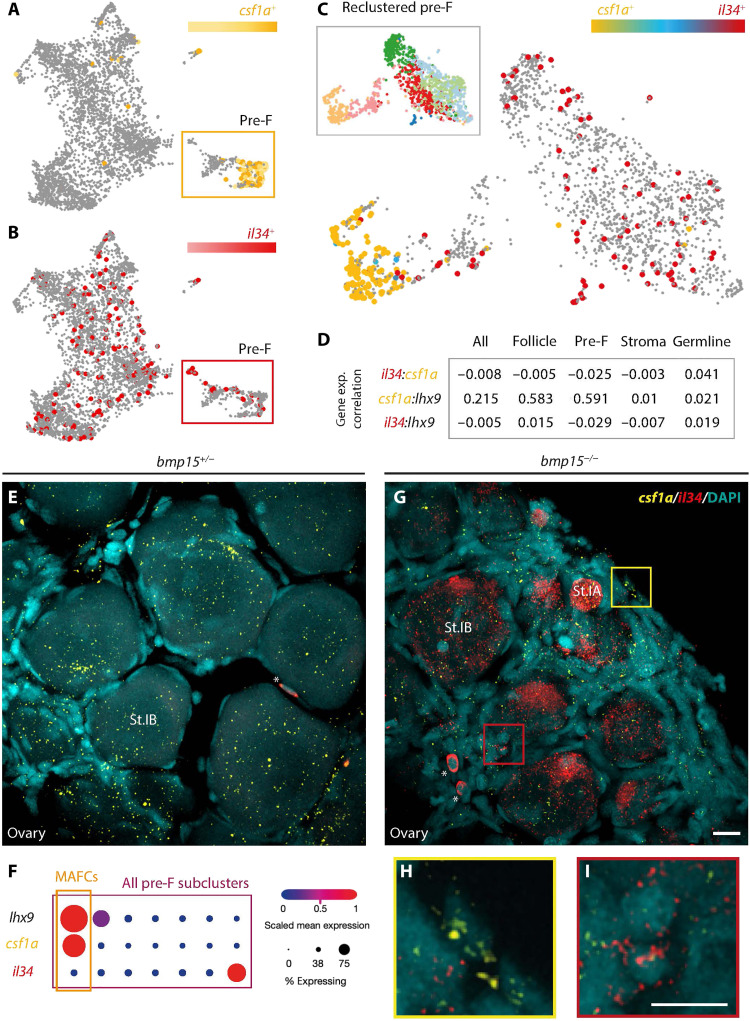
*csf1a* and *il34* are detected in distinct follicle cell populations of the early ovary. (**A** to **C**) UMAP plot of indicated gene’s expression in the 40-dpf ovary in (A) and (B) follicle cells, and (C) reclustered subpopulation of pre-follicle cells. Subpopulation of pre-F cells boxed in (A) and (B). UMAP of reclustered pre-F cells; legend boxed in (C). (**D**) Gene expression correlation values of indicated genes in specified clusters of ovarian cells represented by Spearman’s Rho correlation analysis. (**E** and **G** to **I**) Double HCR RNA FISH maximum projections of confocal images of 40-dpf *bmp15* (E) heterozygous and (G) to (I) mutant ovaries. *il34*, red; *csf1a*, yellow; and DAPI (DNA), cyan. Asterisk (*) indicates blood cells. Regions boxed in (G) are magnified in (H) and (I). Scale bars, 10 μm. (**F**) Analysis of expression profiles of indicated genes in specified clusters of follicle cells represented by dot plot graph. MAFCs, macrophage-activating follicle cells.

Csf1 receptors on macrophages are activated by Csf1 ligands and Il-34 (*45*, *46*); therefore, the cells that express these ligands represent candidate triggers of sex reversal. Thus, we determined which cells in the ovary express RNAs coding for Csf1R-activating ligands. Analysis of reclustered 40-dpf gonadal cell populations, including pre-follicle cell populations, recently defined by scRNA-seq based on their expression of pre-follicle cell genes including *lim homeobox 9* (*lhx9*), *iroquois3a* (*irx3a*), and *iroquois5a* (*irx5a*) (*22*), revealed a unique group of ovarian pre-follicle cells that express *irf8* and *csf1a* ligands (Fig. 4, B to E). Among known *csf1r* ligands, reclustered follicle and pre-follicle populations from 40-dpf scRNA-seq data indicated that *csf1a* RNA is enriched in pre-follicle cells, *interleukin 34* (*il34*) was detected in a distinct population of pre-follicle cells, and *csf1b* was not appreciably expressed (Fig. 4A). On the basis of its limited expression, we reasoned that *csf1b* was not likely the relevant ligand. Using fluorescence in situ hybridization (FISH) to verify expression profiles and spatial distribution of *lhx9*, *csf1a*, and *il34* in 40-dpf wild-type pre-follicle cells, we found that, as indicated by the scRNA-seq data, *csf1a* is expressed in *lhx9-*expressing pre-follicle cells and in *lhx9-*negative cells that are likely stromal cells (Figs. 4, F to H, and 5, A, C, and D). In contrast, although detected in distinct follicle cell populations by single-cell analysis, *il34* was not detectable in wild-type gonads at this stage (Fig. 5, B to F). On the basis of the single-cell data, which indicates that *il34* is most highly expressed in immune cells (Fig. 4A), and the correlation data showing that *il34* is only expressed in relatively few follicle cells (Fig. 5D), *il34*-expressing cells likely become more prominent in the gonad as it differentiates. Analysis of recently published scRNA-seq data of human embryonic ovary (*47*) indicates the presence of *lhx9*-, *csf1a*-, and *irf8-*expressing populations in the human early ovary (fig. S4).

**Fig. 6.**
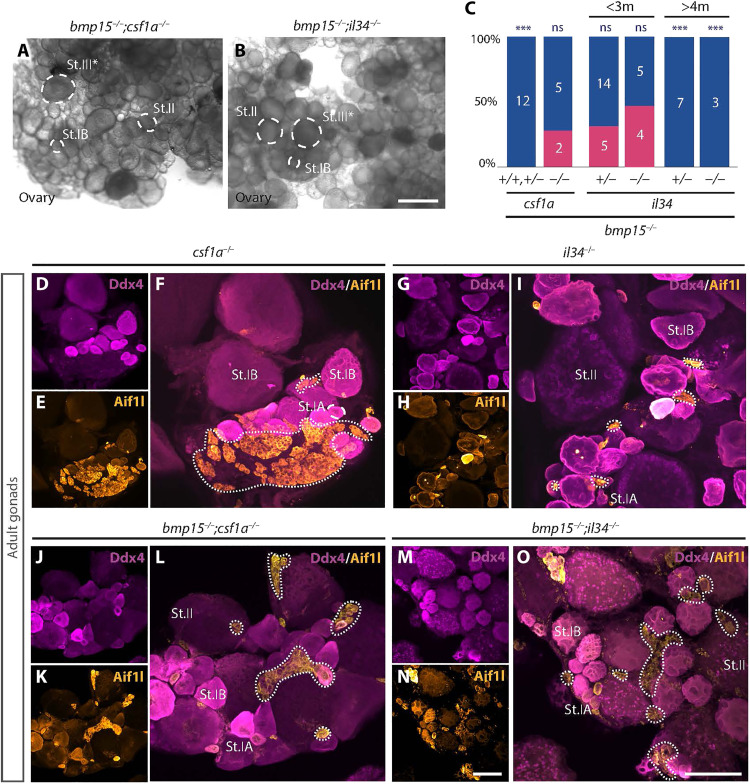
Il34 and Csf1a differentially contribute to ovarian failure and sex reversal and macrophages persist in *csf1a-* and *il34-*mutant ovaries. (**A** and **B**) Live tissue images of adult gonads of indicated genotypes. Scale bar, 500 μm. (**C**) Graph with adult sex ratios of indicated genotypes. Female, pink; male, blue. Numbers indicate individuals examined. Statistical analysis: chi-square test with Bonferroni correction; *P* value comparisons to *bmp15*^+/−^, ***P ≤ 0.0001. (**D** to **O**) Immunostained adult gonads of (E) to (J) ligand single mutants, and (K) to (O) *bmp15;ligand* DMs of indicated genotypes. Aif1l (yellow) labels macrophages, Ddx4 (magenta) labels germ cells, and DAPI (blue) labels DNA. Dotted lines mark macrophages. Scale bars, 100 μm.

To determine whether loss of *bmp15* influences the expression of Csf1Rb ligands, we examined *il34* and *csf1a* in *bmp15-*mutant ovaries. As in wild type (Figs. 4F and 5E), *csf1a* was expressed in distinct subsets of pre-follicle cells but appeared to be more abundant in *bmp15* mutants (Fig. 5, G and H). However, unlike wild-type juvenile ovaries in which *il34* was undetectable (Fig. 5E), *il34* was highly expressed in the germline and somatic cells of *bmp15* mutants (Fig. 5, G and I). Abundant *il34* in *bmp15-*mutant oocytes suggests that Il34 from oocytes could be a trigger of oocyte loss. Although previously thought to signal primarily through Csf1Ra, which is dispensable for ovarian failure and sex reversal on its own, Il34 was recently shown to also signal through Csf1Rb (*48*). Because Csf1a and Il34 ligands can signal through Csf1Rb and that their transcripts were detected in different populations of pre-follicle cells and were elevated in *bmp15* mutants (Fig. 5), it was possible that either ligand could contribute to macrophage activation and ovary-to-testis transition. To determine whether one or both Csf1Rb ligands were required for ovarian atresia and sex reversal, we examined DMs lacking *bmp15* and *csf1a* (*bmp15*;*csf1a DM*s) and DMs lacking *bmp15* and *il34* (*bmp15*;*il34 DM*s). We found that removing Csf1a was sufficient for sustained suppression of ovarian failure and sex reversal of *bmp15* mutants, indicating that Csf1a drives ovary-to-testis transformation (Fig. 6, A and C). Consistent with a role for Il34 in promoting oocyte loss, removal of Il34 suppressed ovarian failure of *bmp15* mutants, but, unlike loss of Csf1a, eliminating Il34 only delayed the ovary-to-testis transition (Fig. 6, B and C). As expected, macrophages marked by Aif1l were still present in *csf1a* single mutants (Fig. 6, D to F), *il34* single mutants (Fig. 6, G to I), *bmp15;csf1aDMs* (Fig. 6, J to L), and *bmp15;il34DMs* (Fig. 6, M to O). Our findings identify a previously unknown role for the subpopulation of pre-follicle cells, hereafter called macrophage-activating follicle cells (MAFCs) based on their expression of *csf1a*, a known ligand for Csf1Rb that is essential for ovary-to-testis transformation. Furthermore, MAFCs are in direct contact with ovarian follicles and thus are positioned to sense oocyte cues and release Csf1a to activate ovarian macrophages.

## DISCUSSION

**Fig. 7.**
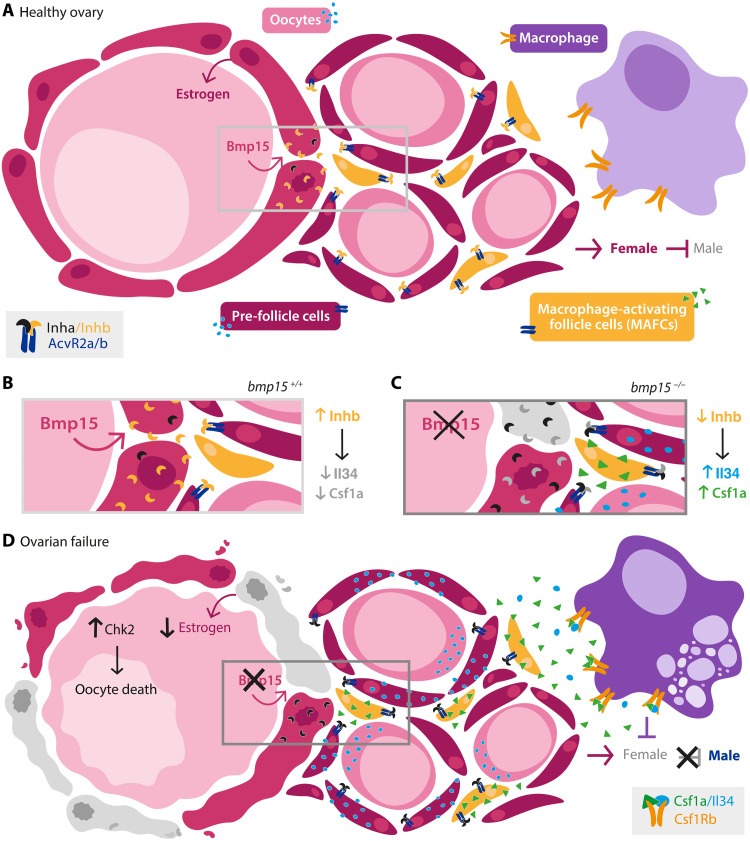
Model of germline-somatic-immune cell axis in healthy ovary and during ovarian failure. Schematic depicts the signals and cellular players in (**A** and **B**) healthy ovary. Bmp15 from the oocyte signals to promote somatic follicle fates and survival such that the balance of Activins (Inhb) and Inhibins (Inha) prevents release of Csf1a and Il34 from pre-follicle cells and MAFCs, which express activin receptors, thereby blocking activation or expansion of macrophages. (**C** and **D**) During ovarian failure caused by loss of Bmp15, this balance tips to favor Inhibin a causing elevated Il34, and MAFCs to release Csf1a. Together, elevated Il34 and Csf1a trigger macrophage activation and activation of programs that regulate ovary-to-testis remodeling and sex reversal in zebrafish. (B) and (C) Enlarged views of regions boxed in (A) and (D).

Although many factors involved in sex determination in zebrafish have been discovered, the molecular trigger initiating sexual differentiation and the factors involved in remodeling the gonad, from juvenile gonad to ovary or testis during development, or sex reversal of adult females during ovarian failure remain elusive (*9*). This work identifies macrophages as mediators of sex reversal during ovarian failure but not during differentiation of the juvenile gonad to testis in zebrafish. Specifically, primitive macrophages, which form before sex determination occurs in zebrafish, are dispensable for ovary-to-testis transition during development and during ovarian failure in adults. In contrast, definitive macrophages peak around the time when sex is determined in zebrafish, suggesting that definitive macrophage development could be influenced by or influence sexual differentiation and the transition from indifferent gonad to ovary or testis during development. However, the observation that adult female:male sex ratios are normal in mutants lacking all macrophages (*csf1r* DMs and *irf8* mutants) indicates that macrophages are not essential triggers of sexual differentiation of the indeterminant gonad nor for development of the juvenile gonad into an ovary or testis. Instead, definitive macrophages are required after sex has been established for ovary-to-testis transformation in response to pathological contexts, like ovarian insufficiency or failure due to mutation of *bmp15.* In this context, macrophages or macrophage activation by MAFCs expressing Csf1a may be the trigger or respond to the trigger for testis differentiation (Fig. 7). This notion is consistent with the observation that loss of Csf1a or definitive macrophages preserves oocytes and blocks ovary-to-testis transition of *bmp15* mutants. Future investigation is needed to determine the developmental roles of MAFCs and how they contribute to testis transformation during ovarian failure in zebrafish.

Ovarian failure in humans has been associated with immunity-related disorders and genetic factors, such as genetic variants in BMP15, but the specific immune cells and mechanisms that drive premature follicle loss, infertility, and masculinization were not known. In zebrafish, as in the mammalian ovary, Bmp15 both regulates cell survival and promotes cell fates needed for follicle progression (*13*–*15*). Failure of *bmp15-*mutant follicles to progress to vitellogenic stages, even when oocyte loss is suppressed by eliminating Chek2, macrophages, or Dmrt1 (*18*), and a recent report showing that loss of Inhibin A (Inha) could both suppress oocyte loss and promote follicle progression through mid-vitellogenic stage underscore the important role of Bmp15 in follicle progression and preservation (*49*). We propose that Bmp15 promotes follicle development and survival and silences specialized pre-follicle cells that express *csf1a* (MAFCs) (Fig. 7). Furthermore, our data suggest that MAFCs may act as sentinels of oocyte or follicle quality. Accordingly, in response to failed follicle differentiation and loss of oocytes or follicle signals, MAFCs would release Csf1a ligand and signal to ovary macrophages to trigger ovary-to-testis sex reversal (Fig. 7).

Given that follicle turnover is an ongoing and normal process in ovaries, there must be mechanisms to control macrophage activity and responses to prevent widespread ovarian failure. The finding that both Csf1a and Il34 ligands contribute to ovary-to-testis transformation associated with ovarian failure is exciting because it provides a mechanism for differential macrophage activation that could allow macrophages within the ovary to distinguish between normal homeostatic turnover or “quality control” and catastrophic events within the germline/follicle, such as loss of Bmp15. While Csf1a is required and its loss completely blocks ovary-to-testis transition, loss of Il34 only delays it, suggesting that the two ligands contribute differently and that Csf1a is the main driver of ovary-to-testis transformation during ovarian failure. That these ligands contribute distinctly is expected because single-cell and spatial transcript analyses show that they are expressed in different subsets of cells within the ovary. Moreover, in other contexts, macrophage stimulation by Csf1a versus Il34 has been shown to have different consequences on macrophage activation; for example, Csf1 has been reported to induce molecular signatures associated with enhanced phagocytic activity, aggregation and migration, while Il34 does not or does so to a lesser degree (*50*). Further investigation will be required to understand the specific roles of Csf1a and Il34 and to determine whether the distinct contributions of these ligands are solely due to their expression profiles or instead reflect differences in their activities, unique roles of their respective pre-follicle cell populations, or a combination of these factors. Conservation of the molecular pathways and their respective reproductive and immune functions suggests that our discoveries and the arising questions may more broadly represent cellular and molecular targets to prevent premature oocyte loss or ameliorate aspects of ovarian insufficiency/failure-related reproductive disorders.

In addition to premature ovarian insufficiency due to genetic factors, POI and permanent infertility are serious side effects of chemotherapy that affect reproductive health and general health more broadly of young cancer survivors, and there is no standard of care to preserve ovarian health after chemotherapy (*51*). Moreover, Csf1 is elevated in numerous cancers, including reproductive cancers; thus, elevated Csf1 and macrophage dysregulation might similarly contribute to adverse effects on fertility in this context (*50*). Our finding that eliminating Csf1 allows for sustained maintenance of oocytes and prevents ovarian failure due to genetic factors raises the possibility that blocking Csf1 might be a strategy to preserve ovarian health during chemotherapy.

## MATERIALS AND METHODS

### Animals

Mutant zebrafish lines used were *bmp15^uc31^*, *chek2^sa20350^*, *csf1a^re05^*, *csf1ra^j1e4^*, *csf1rb^re01^*, *il34^re07^*, *irf8^st96^*, *tp53^zdf1^*, and Tg(*piwil1:egfp*)*^uc02^* (*7*, *37*, *40*, *43*, *45*, *47*, *52*). Experimental fish used for whole-mount immunohistochemistry and live tissue images were 3 months old or older (referred in the figure legends as “adults”). Fish used for scRNA-seq libraries and single-molecule whole-mount RNA FISH were 40 dpf. Standard conditions were used for maintenance of all zebrafish. All protocols and procedures were performed following the guidelines from the National Institutes of Health and approved by the Icahn School of Medicine at Mount Sinai Institutional (ISMMS) Animal Care and Use Committees (IACUC, 2017-0114).

### Genotyping

Samples from fin clip, trunk, or gonad were lysed in an alkaline lysis buffer [25 mM NaOH and 0.2 mM EDTA (pH 12)] to obtain genomic DNA (gDNA), heated at 95°C for 20 min, and cooled at 4°C before adding neutralization buffer [20 mM tris-HCl and 0.1 mM EDTA (pH 8.1)] (*53*). gDNA was polymerase chain reaction (PCR)–amplified, followed, when needed, by restriction enzyme digestion, and then resolved in a 3% 1:1 MetaPhor/agarose gel (primers and restriction enzymes used for each gene listed in table S1).

### Dissections

Fish were anesthetized with a lethal dose of tricaine (MS-22) (400 mg/liter). Sex was assessed by imaging overall body morphology, tubercle morphology (or lack thereof on pectoral fins), and urogenital papilla morphology. Gonads were dissected before or after fixation. Briefly, the head was removed with a razorblade; then, using forceps, the body was opened along the ventral body wall (anterior to posterior). Gastrointestinal organs and swim bladder were removed to expose the gonad (ovary/testis/indeterminant). Both lobes of the gonad were dissected out of the body cavity. Images were acquired of intact and gently dissociated gonads to confirm the sex of the fish. Dissected gonads were subsequently fixed in 4% paraformaldehyde (PFA) overnight and then dehydrated in 100% methanol (MeOH). All live anatomical tissue images were acquired with a Zeiss Stemi stereo microscope. Dissected gonads were imaged using a Zeiss Zoom stereo or fluorescence microscope.

### Single-molecule whole-mount RNA in situ hybridization

Fish trunks were fixed overnight at 4°C in 4% PFA and washed with phosphate-buffered saline (PBS), dehydrated with MeOH, and then placed at −20°C overnight or until use. HCR RNA FISH probes were designed from Molecular Instruments (table S2). Hybridization was performed following the manufacturer’s protocol (MI-Protocol-RNAFISH-Zebrafish) with the following changes: After rehydration in PBS with a series of MeOH>PBS 5-min washes, gonads were dissected and washed four times for 5 min in PBST (PBS and 1% Tween 20). Gonads were permeabilized with proteinase K at 50 μg/ml in PBST for 15 min. After the last manufacturer’s protocol step, samples were cleared in 1 hour of incubations of 30, 50, and 70% glycerol/PBS. Last, samples were mounted in ProLong Diamond Antifade Mountant with 4′,6-diamidino-2-phenylindole (DAPI) (Invitrogen) and imaged with a confocal microscope Zeiss 980 AiryScan2.0 (63× oil objective, 1024 × 1024 pixel format). All pictures were processed using ImageJ/Fiji.

### Whole-mount immunofluorescence

Tissues were fixed with 4% PFA overnight at 4°C and washed the next day in PBS before dehydration with MeOH. Samples were stored at −20°C for at least one night or until use. Tissues were rehydrated with several washes of PBS before permeabilization with acetone for 10 min at −20°C and then incubated with (i) blocking buffer (5% normal goat serum/2% dimethyl sulfoxide in 0.1% Tween 20/PBS) at room temperature for 1 hour or at 4°C overnight and (ii) primary antibody overnight at 4°C; (iii) washed in PBS plus 0.05% Tween 20 (PBST); (iv) incubated in secondary antibody at room temperature for 2 hours or overnight at 4°C; and (v) washed in PBST. To label germ cells, we used a chicken anti-Ddx4 primary antibody (*54*) at a 1:3000 dilution (table S2) followed by Alexa Fluor 488 or Alexa Fluor 647 secondary antibody (Molecular Probes) diluted 1:500. To label macrophages, we used a rabbit anti-Aif1l or mouse anti-4c4 primary antibody (*34*, *36*) at a 1:200 dilution (table S2) followed by Alexa Fluor 568 or Alexa Fluor 680 secondary antibody (Molecular Probes) diluted 1:500. Whole-mount tissues were mounted on slides using Vectashield with DAPI (Vector Laboratories) or ProLong Diamond Antifade Mountant with DAPI (Invitrogen) and imaged using a Zeiss Axio Observer inverted microscope equipped with Apotome.2 and a charged-coupled device camera or a confocal microscope Zeiss 980 AiryScan2.0 (20× objective, 1024 × 1024 pixel format). All pictures were processed using ImageJ/Fiji and Adobe Illustrator.

### Single-cell RNA sequencing

scRNA-seq library expression data were from raw and processed data obtained from (*43*) for the zebrafish ovary and from (*46*) for the fetal human ovary. Uniform manifold approximation and projection (UMAP) plots (UMAPS) and graphs were generated using BBrowser3 software and online browsers embedded in the zebrafish (Single Cell Portal) and human (CellxGene) publications, following the published analysis parameters.

### Statistical analysis

Statistical analysis of sex ratio comparisons of all mutants analyzed was performed by Chi-square test with Bonferroni correction. *P* value comparisons were made between *bmp15-*mutant genotypes and *bmp15*^+/−^ genotypes, *P ≤ 0.0125 and ***P ≤ 0.0001.
