# Supplementary material for: Macrophage activation drives ovarian failure and masculinization in zebrafish
Source: Sci Adv. 2023 Nov 22;9(47):eadg7488. doi: 10.1126/sciadv.adg7488 (PMC10664988; doi:10.1126/sciadv.adg7488)
Supplement: Supplementary file 1 — Figs. S1 to S4 Tables S1 and S2 [file sciadv.adg7488_sm.pdf]

Science Advances
AAAS

Supplementary Materials for

Macrophage activation drives ovarian failure and masculinization in zebrafish

Paloma Bravo *et al.*

Corresponding author: Florence L. Marlow, florence.marlow@mssm.edu

Sci. Adv. **9**, eadg7488 (2023)
DOI: 10.1126/sciadv.adg7488

This PDF file includes:

Figs. S1 to S4
Tables S1 and S2

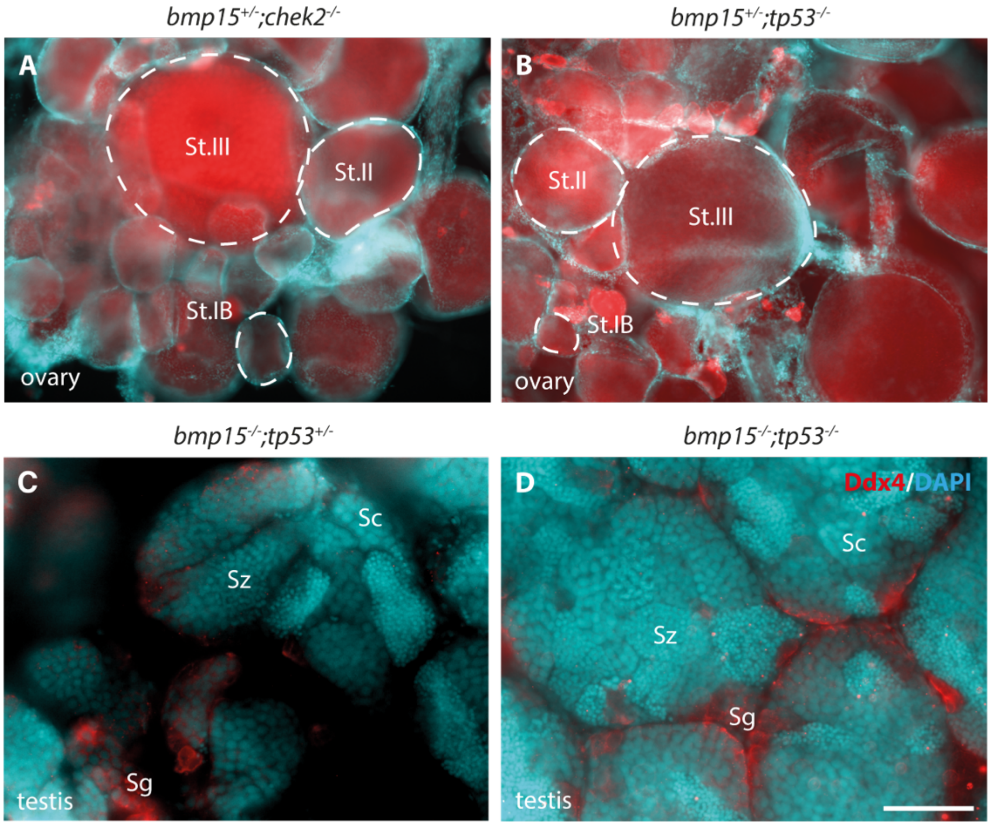

Fig. S1.

Chek2 and Tp53 are dispensable for sex determination and differentiation. (A-D) Immunostained adult gonads of indicated genotypes. Ddx4 (red) labels germ cells, DAPI (cyan) labels DNA. Scale bar: (A-B) 250µm, (C-D) 50µm. St. IB: stage IB oocyte (late prophase within definitive follicles), St.II: stage II oocyte (apparent cortical alveoli and a vitelline envelope), St. III: stage III oocyte (increased size and apparent yolk). Sg: spermatogonia, Sc: spermatocyte, Sz: spermatozoa.

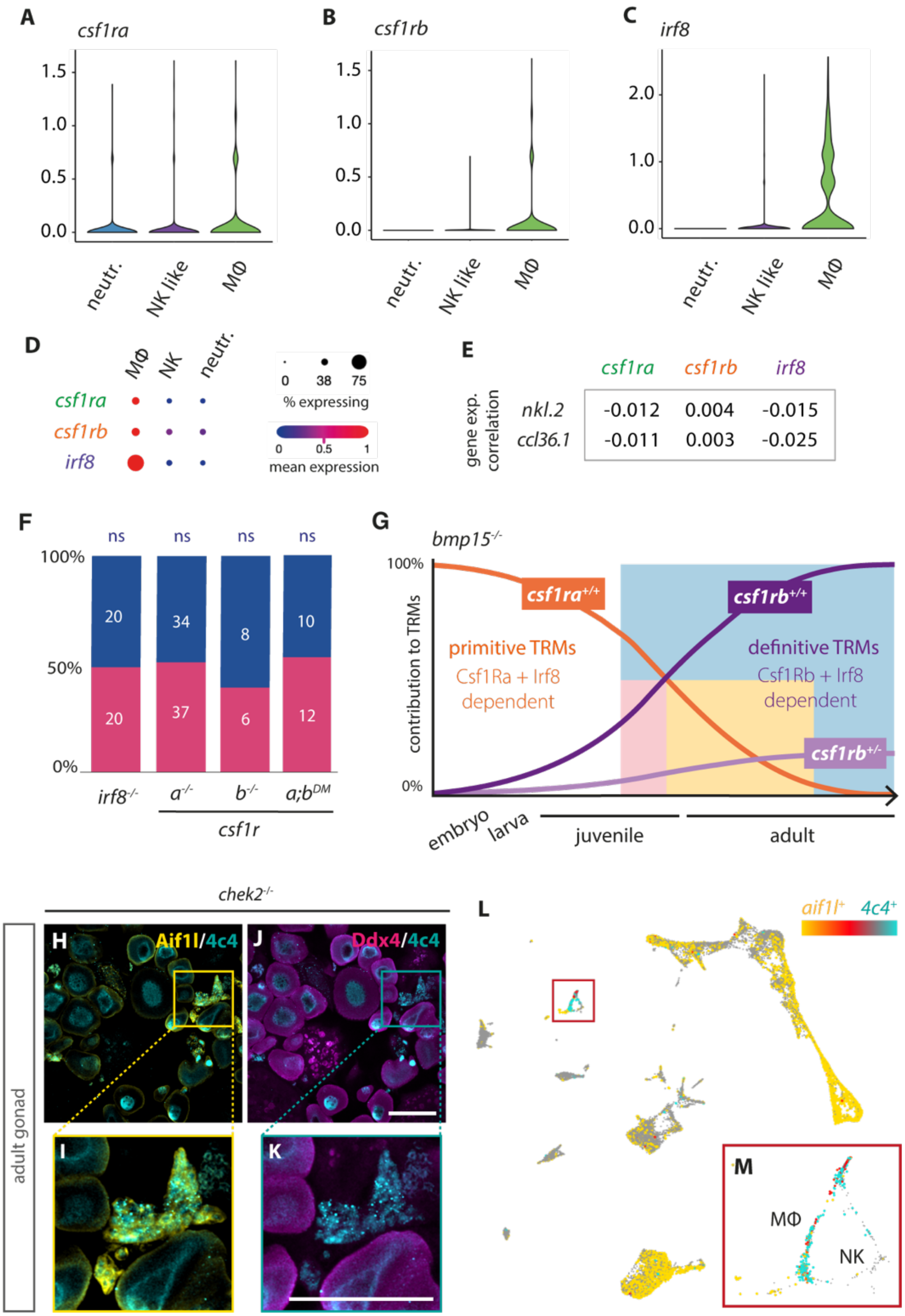
A
csf1ra
B
csf1rb
C
irf8
neutr.
NK like
MΦ
D
MΦ
NK
neutr.
csf1ra
csf1rb
irf8
0 38 75
% expressing
0 0.5 1
mean expression
E
gene exp. correlation
csf1ra csf1rb irf8
nkl.2 -0.012 0.004 -0.015
ccl36.1 -0.011 0.003 -0.025
F
ns
100%
50%
0%
irf8-/-
a-/-
b-/-
a;bDM
csf1r
G
bmp15-/-
contribution to TRMs
csf1ra+/+
csf1rb+/+
csf1rb+/-
primitive TRMs
Csf1Ra + Irf8
dependent
definitive TRMs
Csf1Rb + Irf8
dependent
embryo
larva
juvenile
adult
chek2-/-
adult gonad
H
Aif1l/4c4
J
Ddx4/4c4
I
K
L
aif1l+ 4c4+
M
MΦ
NK

Fig. S2.

Macrophages are present in early ovary but are not required for normal sex determination or differentiation. (A-D) Expression of indicated genes in different immune cell populations represented by (A-C) violin and (D) dot plot graphs. MΦ: macrophages, NK: natural killer-like cells, neutr.: neutrophils. **(E)** Analysis of expression profiles of indicated genes in specified clusters of immune cells represented by Spearman's Rho correlation values. **(F)** Adult sex ratios of indicated genotypes. Female (pink), male (blue). Numbers indicate individuals examined. Statistical analysis: Chi-square test with Bonferroni correction; p-Value comparisons to *bmp15*$^{+/}$. **(G)** Diagram representation of macrophage waves specification and contribution in zebrafish. Sex after differentiation represented by background color: pink, female; blue, male; yellow, sex-reversal. X-axis: fish stage, y-axis: percentage of macrophage. **(H-K)** Immunostained adult gonads of indicated genotype. Aif1l (yellow) and 4c4 (cyan) label macrophages, Ddx4 (magenta) labels germ cells. (I, K) Magnified view boxed in (H, J). Scale bar: 100µm. **(L-M)** UMAP plots showing co-expression of indicated genes in the 40dpf ovary. (M) Magnified population of immune cells boxed in (L).

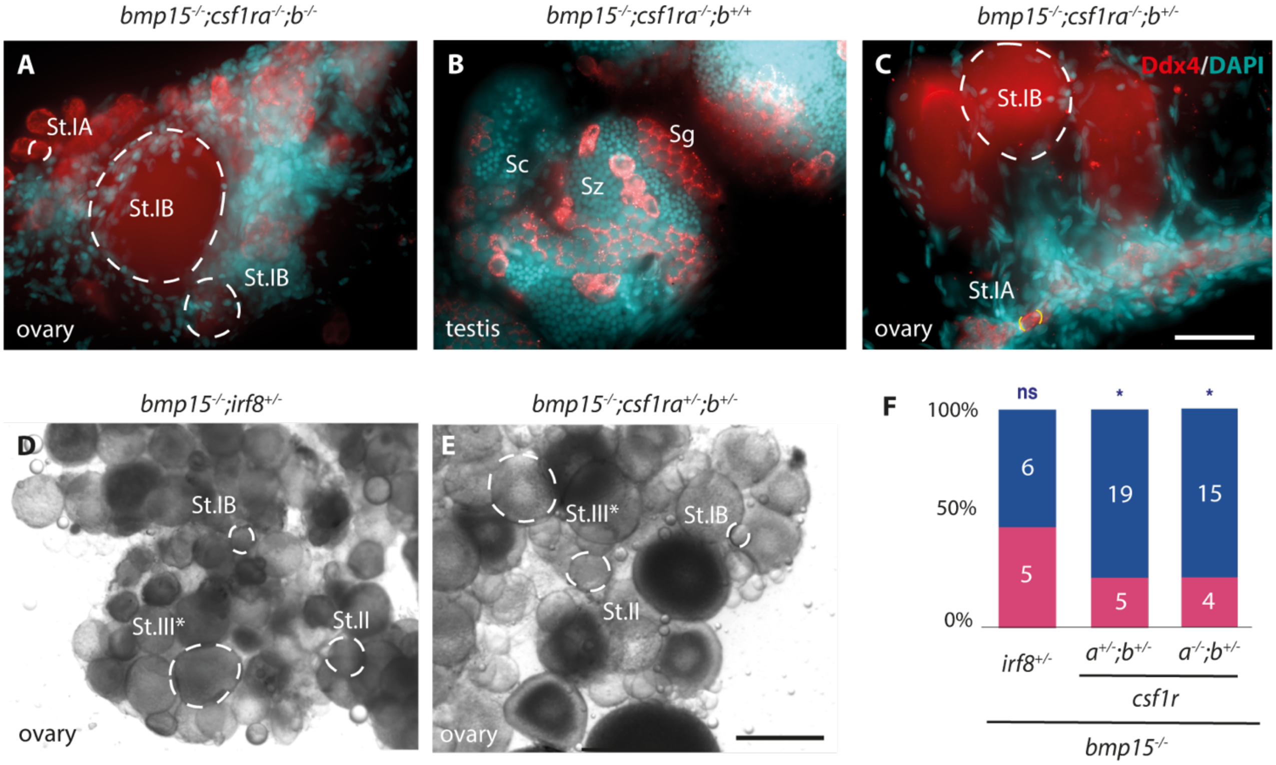

Fig. S3.

Definitive macrophages are required for ovarian failure and sex reversal of *bmp15* mutant females. (A-C) Immunostained adult *bmp15* mutant gonads lacking (A) all, (B) only primitive, or (C) primitive and haploinsufficiency for definitive macrophages. Ddx4 (red) labels germ cells, DAPI (cyan) labels DNA. Scale bar: 50µm. St. IB: stage IB oocyte (late prophase within definitive follicles), St.II: stage II oocyte (apparent cortical alveoli and a vitelline envelope), St. III*: arrested stage III oocyte (increased size and apparent yolk), Sg: spermatogonia, Sc: spermatocyte, Sz: spermatozoa. **(D-E)** Live tissue pictures of adult gonads of *bmp15* mutant fish heterozygous for (D) *irf8* or (E) *csf1rs*. Scale bar: 500 µm. **(F)** Adult sex ratios graph of indicated genotypes. Female (pink); male (blue). Numbers indicate individuals examined. Statistical analysis: Chi-square test with Bonferroni correction; p-Value comparisons to *bmp15*$^{+/-}$, *P $\leq$ 0.0125.

Fig. S4.

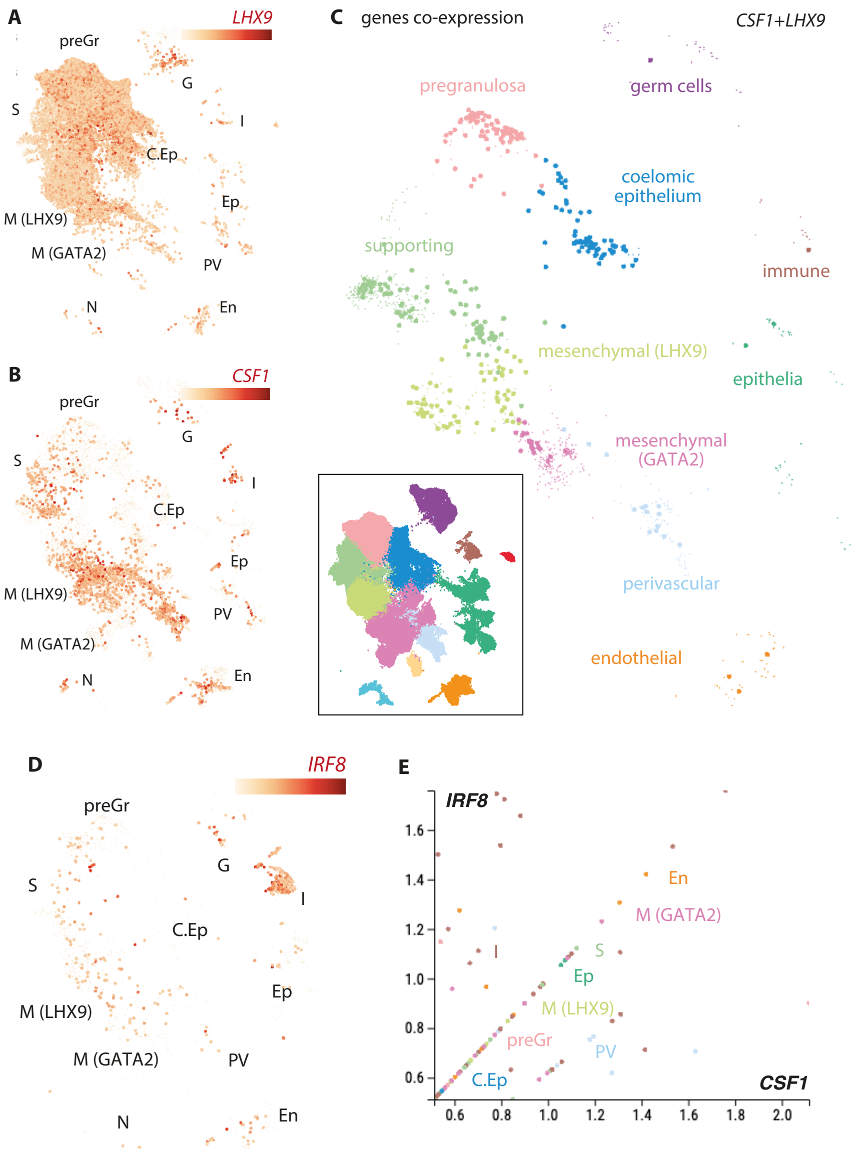

CSF1 source cells in human fetal ovary. (A-D) UMAP plots of indicated gene expression profiles in the fetal human ovary showing (A, B, D) overall expression and (C) co-expression. UMAP clusters legend boxed in (C). **(E)** Scatterplot of cell clusters co-expressing indicated genes. C.Ep: coelomic epithelium, En: endothelial, Ep: epithelia, I: immune, M: mesenchymal, preGr, pregranulosa, PV: perivascular, S: supporting.

Gene	Allele	F primer	R primer	RE (cuts)	source
bmp15	***uc31***	AGCCTTTCAGGTGGCACTCG	CCACTGAAAAACACTTTCTCCC	-	*(51)*
chek2	***sa20350***	AGCCACACGAAATGCTGAG	CAGACTGAAGACTCCTACTACATTG	HpyCH4III (mut)	ZIRC
csf1a	***re05***	GCCGGTTGAGCTTCTGAAAAT	GCATTTTGGTTAGGCTGCTG	-	*(43)*
csf1ra	***j4e1***	TCTGGGCAAAGAGGACAACATCACAC	CCACAGCTCTGCAAGGTTTG	SpeI (wt)	*(52)*
csf1rb	***re01***	GGACAGAGTTTTCGCTCCAG	ATTGGACTCCGCTCATGTTC	MspI (wt)	*(37)*
il34	***re07***	TGGTCTTCGTGATTCCCTTC	TGCTCCTCATTCCTTCAACC	-	*(43)*
irf8	***st96***	ACATAAGGCGTAGAGATTGGACG	GAAACATAGTGCGGTCCTCATCC	AvaI (wt)	*(40)*
tp53	***zdf1***	ACATGAAATTGCCAGAGTATGTGTC	TCGGATAGCCTAGTGCGAGC	-	*(53)*

Table S1.
Experimental models and genotyping assays.

Name	[conc]	use	source (catalog #)
Chicken anti-Ddx4	1:3000	IHC	PMID: 30653507
csf1a	8nM	FISH	Molecular Instruments (custom)
il34	8nM	FISH	Molecular Instruments (custom)
Rabbit anti-Aif1l (Iba1)	1:200	IHC	FUJIFILM Wako Chemicals
Mouse anti-Y14 (4c4)	1:200	IHC	EMD Millipore Corp (05-1511)
lhx9	8nM	FISH	Molecular Instruments (PRD333)

Table S2.
Antibody and probes for fluorescence staining.
